# Alfalfa growth and nitrogen fixation constraints in salt-affected soils are in part offset by increased nitrogen supply

**DOI:** 10.3389/fpls.2023.1126017

**Published:** 2023-02-21

**Authors:** Weifan Wan, Qian Liu, Caihong Zhang, Ke Li, Zhi Sun, Yuejin Li, Haigang Li

**Affiliations:** Inner Mongolia Key Laboratory of Soil Quality and Nutrient Resources, Key Laboratory of Agricultural Ecological Security and Green Development at Universities of Inner Mongolia Autonomous Region, Inner Mongolia Agricultural University, Hohhot, China

**Keywords:** alfalfa, salt stress, yield, N uptake, quality, N fixation

## Abstract

**Introduction:**

In China, alfalfa (*Medicago sativa* L.) is often grown on marginal land with poor soil fertility and suboptimal climate conditions. Soil salt stress is one of the most limiting factors for alfalfa yield and quality, through its inhibition of nitrogen (N) uptake and N fixation.

**Methods:**

To understand if N supply could improve alfalfa yield and quality through increasing N uptake in salt-affected soils, a hydroponic experiment and a soil experiment were conducted. Alfalfa growth and N fixation were evaluated in response to different salt levels and N supply levels.

**Results and discussion:**

The results showed that salt stress not only significantly decreased alfalfa biomass, by 43%–86%, and N content, by 58%–91%, but also reduced N fixation ability and N derived from the atmosphere (%Ndfa) through the inhibition of nodule formation and N fixation efficiency when the salt level was above 100 mmol Na_2_SO_4_ L^–1^. Salt stress also decreased alfalfa crude protein by 31%–37%. However, N supply significantly improved shoot dry weight by 40%–45%, root dry weight by 23%–29%, and shoot N content by 10%–28% for alfalfa grown in salt-affected soil. The N supply was also beneficial for the %Ndfa and N fixation for alfalfa with salt stress, and the increase reached 47% and 60%, respectively. Nitrogen supply offset the negative effects on alfalfa growth and N fixation caused by salt stress, in part through improving plant N nutrition status. Our results suggest that optimal N fertilizer application is essential to alleviate the loss of growth and N fixation in alfalfa in salt-affected soils.

## 1 Introduction

Alfalfa (*Medicago sativa* L.) is a forage crop with high yield capacity, good palatability, and high nutritive value, and is widely cultivated across the world (Koenig et al., 1999). However, alfalfa is often sown in marginal land in China, especially in suboptimal climate conditions and salt-affected soils, which usually have poor soil fertility (Jia et al., 2006; Fan et al., 2016). Salt-affected soils account for 25% of farmland (99.13 million hectares) in China and are mainly distributed in arid and semiarid areas (Zhao and Li, 1999). Thus, salinity is one of the major limiting factors for alfalfa productivity in these areas (Peel et al., 2004; Nadeem et al., 2019). Strong evaporation in spring accelerates salinization processes in the top layer of salt-affected soils and creates a serious stress for alfalfa regrowth and emergence (Guan et al., 2019).

As a legume, alfalfa is more sensitive to salt stress than cereals (Isayenkov, 2012). Previous studies have shown that salt stress significantly reduces alfalfa germination by weakening respiration, reduces biomass production by inhibiting photosynthesis, and reduces forage quality by decreasing soluble protein (Esechie et al., 2002; Dong et al., 2018; Lu et al., 2021). Moreover, salt stress also reduces the nutrient adsorption ability of plants, including nitrogen (N) adsorption ability, indirectly decreasing plant growth (Zhu, 2001). This decline in N uptake, translocation, and metabolism has been observed in soybeans (*Glycine max* L.) experiencing salt stress (Ghassemi-Golezani et al., 2010). This is due to the decrease in N accumulation caused by a low adsorption rate of ${\text{NH}}_{4}^{\;+}$ and ${\text{NO}}_{3}^{\;-}$ (Frechilla et al., 2001).

N fixation is a source of N acquisition in legumes. The percentage of alfalfa N fixation from air to total N uptake reaches 83% uptake during the growing season (Burity et al., 1989). Salt stress can significantly reduce N fixation by inhibiting the germination of rhizobium–legume symbioses (Del Pilar Cordovilla et al., 1999), inducing the deformation of root hairs (Zahran, 1999), reducing the nitrogenase activity of nodules (Fahmi et al., 2011), and disturbing signal exchange processes (Miransari and Smith, 2007). The decrease in N fixation efficiency is also ascribed to the decline in leghemoglobin content, respiration rate, malate concentrations in nodules, and photosynthate availability (Swaraj and Bishnoi, 1999). Raun et al. (1999) found that N fertilizer application (< 50 kg ha^–1^) improves nodule formation and biological N fixation efficiency.

The N supply also increases the activity of defense enzymes and promotes N metabolism in plants suffering salt stress (Kirova, 2020; The et al., 2021). In addition, N supply increases alfalfa crude protein (CP), and decreases acid detergent fiber (ADF) and neutral detergent fiber (NDF) (Slamet et al., 2012). However, the response of alfalfa growth and N fixation to N supply under salt stress conditions is unclear. Therefore, we hypothesized that N supply can improve plant N nutrition and offset the negative effects of salt stress on alfalfa growth and N fixation. The objectives of this study were to (1) evaluate alfalfa growth and N fixation in response to different salt levels; and (2) assess the effect of N supply on alfalfa growth and N fixation in salt-affected soil.

## 2 Materials and methods

### 2.1 Experimental set-up

#### 2.1.1 Experiment 1

To test the response of alfalfa growth and N fixation to salt stress, a hydroponic experiment was conducted with five salt levels of 0, 50, 100, 150 and 200 mmol Na_2_SO_4_ L^–1^. There were four replicates in each treatment. The alfalfa seeds (*Medicago sativa* L. cv. Zhongmu No. 1) were surface sterilized with 10% H_2_O_2_ for 30 min. After being rinsed thoroughly in deionized water, seeds were pre-germinated on filter papers in the dark at 25°C. When root length reached 2 cm, six seedlings were transplanted into each pot, which contained 2 L of a nutrient solution. The nutrient solution consisted of (in mmol L^−1^) NH_4_NO_3_ 5, K_2_SO_4_ 0.7, CaCl_2_·2H_2_O 1.65, and MgSO_4_·7H_2_O 1; and (in μmol L^−1^) Fe 10 as EDTAFe-Na, Mn 6 as MnSO_4_·H_2_O, Zn 6 as ZnSO_4_·7H_2_O, Cu 1 as CuSO_4_·5H_2_O, B 4 as H_3_BO_3_, and Mo 1 as (NH_4_)_6_Mo_7_O_4_·4H_2_O. The pH of the nutrient solution was adjusted to 6.5 every day and replaced every 5 days. Plants grew in a phytotron with a light/dark regime of 14/10 hours, relative air humidity of 45%–55%, and an average temperature of 25°C. An additional four pots of wheat (*Triticum aestivum* L. cv. Neimai No. 18) were included as non-N-fixing reference plants for the calculation of N derived from the atmosphere (%Ndfa). All treatments were harvested at 62 days after transplanting.

#### 2.1.2 Experiment 2

To test the effect of N supply on alfalfa growth and N fixation under salt stress, a soil experiment with three N supply rates and two salt levels was set up. The N supply rates were 0, 100, and 200 mg N kg^–1^ as (NH_4_)_2_SO_4_ and salt levels were 0 and 7.1 g Na_2_SO_4_ kg^–1^, which is equal to 50 mmol Na_2_SO_4_ L^–1^ in a hydroponic culture. Each treatment was replicated four times. The soil was collected from the top layer (0–20 cm) in a field at Hailiutu Research Base, Hohhot, China (40°38′N, 111°28′E). The soil was calcareous alkaline soil with pH 8.85, Electrical Conductivity of a saturated soil Extract (ECe) 130.2 µS cm^–1^, Olsen phosphorus 2.61 mg kg^–1^, NH_4_OAc-K 95.37 mg kg^–1^, ${\text{NH}}_{4}^{+}-\text{N}$ 1.5 mg kg^–1^, and ${\text{NO}}_{3}^{-}-\text{N}$ 5.0 mg kg^–1^. After being air dried, the soils were sieved at 2 mm. Basal nutrients were added into soil at the following rates (μg g^–1^): KH_2_PO_4_ 100, K_2_SO_4_ 271.88, MnSO_4_·H_2_O 12.29, ZnSO_4_·7H_2_O 8.86, CuSO_4_·5H_2_O 1.95, Na_2_MoO_4_·2H_2_O 1.01, and FeNaEDTA·3H_2_O 37.60. The same cultivar of alfalfa and germination process were used in this experiment as in experiment 1. After 2 days of germination, 10 seeds were sown in each pot and thinned to six at 7 days after sowing (DAS). Soil moisture in pots was maintained at 70% field capacity by weighing. Plants were harvested at 58 DAS. The same wheat cultivar was sowed as non-N-fixing reference plants for the calculation of %Ndfa.

### 2.2 Plant harvest and analyses

At harvest, the shoots were cut off at the soil surface. All the roots were carefully collected. Roots were shaken to remove loose soil and then submerged in water to remove the attached soil. The alfalfa height, branching, stem diameter, and nodules were recorded. Root and shoot samples were dried in an oven at 70°C for 72 h and weighed.

Shoot δ^15^N and N concentration were measured using an isotope facility (Iso- prime100, Elementar, Germany). Root N concentration was measured using an elemental analyzer (Elementar vario MACRO cube, Germany). The %Ndfa of alfalfa was calculated following the equation: %Ndfa = (δ^15^Nreference plant − δ^15^Nalfalfa/δ^15^Nreference plant − β) × 100, where β is the δ^15^N of alfalfa when wholly reliant on N fixation for its N nutrition (Fan et al., 2006). The amount of N fixed by the alfalfa was calculated using the following equation: N fixed = %Ndfa (%) × shoot dry weight × shoot N concentration (%). The N fixation efficiency of nodules was calculated following the following equation: N fixation efficiency = amount of N fixed/nodule weight (Döbereiner, 1966).

The CP content was determined by a laboratory N concentration analysis, from which the CP content can be calculated by multiplying the N concentration by 100/16, or 6.25 (Mulder, 1839). The NDF and ADF contents were determined by the Van Soest method (Van Soest et al., 1991).

### 2.3 Statistical analysis

All parameters were analyzed using analysis of variance by SAS (v8, SAS Institute Inc., Cary, NC, USA). When effects were statistically significant, the least significant difference (LSD) at *p* = 0.05 is presented. Figures were produced in SigmaPolt software (v10.0, Systat Software, San Jose, CA, USA).

## 3 Results

### 3.1 Alfalfa growth

**Figure 1:**
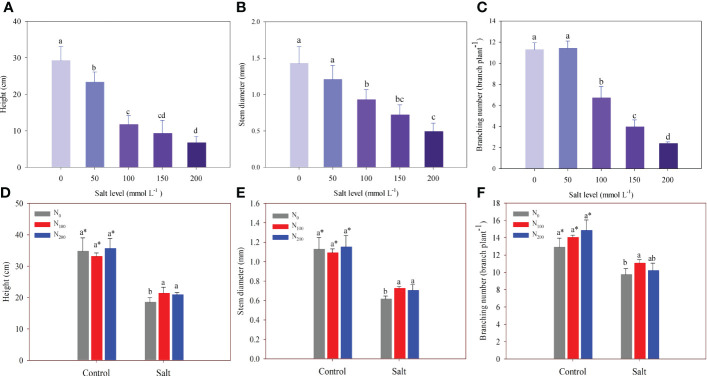
Alfalfa height **(A)**, stem diameter **(B)**, and branching number **(C)** in different salt levels in experiment 1. Alfalfa height **(D)**, stem diameter **(E)**, and branching number **(F)** in different nitrogen (N) supply and salt levels in experiment 2. Error bars represent ± SD of the mean. Different letters represent a significant difference among treatments (*p* ≤ 0.05). Asterisks refer to significant differences between salt levels at the same N fertilizer supply rates.

In experiment 1, alfalfa growth decreased significantly with increasing salt levels (**Figures 1A–C**). The decrease in alfalfa height ranged from 20.1% to 77.1% in salt treatments compared with the control (**Figure 1A**). Stem diameters of alfalfa in 100, 150, and 200 mmol Na_2_SO_4_ L^–1^ levels were significantly lower than those in the control by 35.1%, 49.9%, and 66.0%, respectively (**Figure 1B**). However, a salt level of 50 mmol L^–1^ did not change stem diameter compared with the control. Branching number decreased significantly when the salt level was above 100 mmol L^–1^. The fewest branches were observed in the treatment of 200 mmol Na_2_SO_4_ L^–1^, which produced 2.38 branches per plant (**Figure 1C**).

In experiment 2, although plant height, stem diameter, and branching were significantly inhibited by salt, N supply partially countered these effects (**Figures 1D–F**). In contrast, N supply did not improve the plant height, stem diameter, or branching of alfalfa when compared with alfalfa that was not treated with N supply and did not suffer salt stress. Plant height and stem diameter were higher (13%–18%) in alfalfa with N supply treatment than in alfalfa with no N supply that suffered salt stress (**Figures 1D, E**). There was no difference in plant height and stem diameter between the alfalfa treated with 100 and 200 mg N kg_-1_ supply. However, an increase in branching was observed only in the alfalfa treated with 100 mg N kg^–1^ supply, which was higher by 13% than in the alfalfa treated with no N supply (**Figure 1F**).

**Figure 2:**
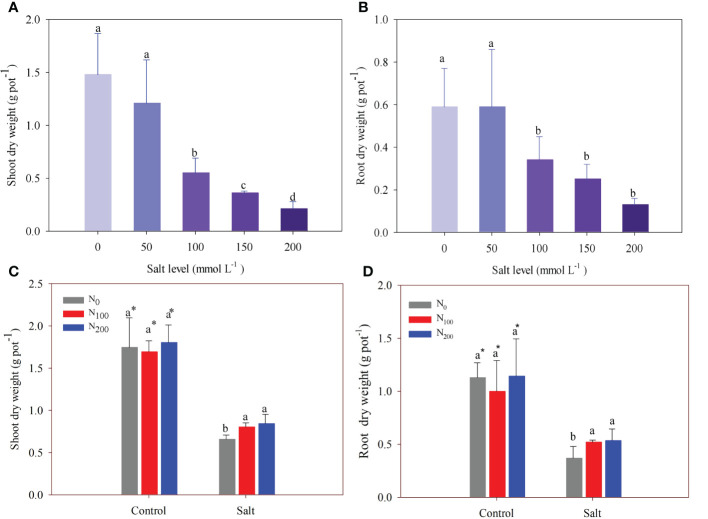
Alfalfa shoot **(A)** and root **(B)** dry weight in different salt levels in experiment 1. Alfalfa shoot **(C)** and root **(D)** dry weight in different nitrogen (N) supply and salt levels in experiment 2. Error bars represent ± SD of the mean. Different letters represent a significant difference among treatments (*p* ≤ 0.05). Asterisks refer to significant differences between salt levels at the same N fertilizer supply rates.

In experiment 1, there was no difference in shoot and root dry weight between the control and the treatment of 50 mmol Na_2_SO_4_ L^–1^(**Figures 2A, B**). However, salt addition at levels of 100, 150, and 200 mmol Na_2_SO_4_ L^–1^significantly decreased shoot dry weight compared with the control by 63%, 76%, and 86%, respectively (**Figure 2A**). Like shoot dry weight, root dry weight decreased by 43%, 53%, and 79% under the same treatments, respectively. Alfalfa root dry weight did not significantly further decrease when salt exceeded 100 mmol Na_2_SO_4_ L^–1^ (**Figure 2B**).

In experiment 2, N supply did not change shoot and root dry weight in alfalfa treated without salt stress (**Figures 2C, D**), whereas salt stress significantly decreased shoot and root dry weight by 49%–67% when compared with treatments without salt stress. In alfalfa treated with N supply, shoot and root dry weights were greater than in alfalfa without N supply when the plants suffered salt stress (**Figures 2C, D**). Shoot dry weight significantly increased by 40% and 45% in alfalfa treated with 100 and 200 mg N kg^–1^, respectively, compared with alfalfa treated without N supply (**Figure 2C**). Root dry weight significantly increased by 23% and 29% in alfalfa treated with 100 and 200 mg N kg^–1^, respectively, compared with alfalfa treated without N supply (**Figure 2D**).

### 3.2 Alfalfa N uptake

**Figure 3:**
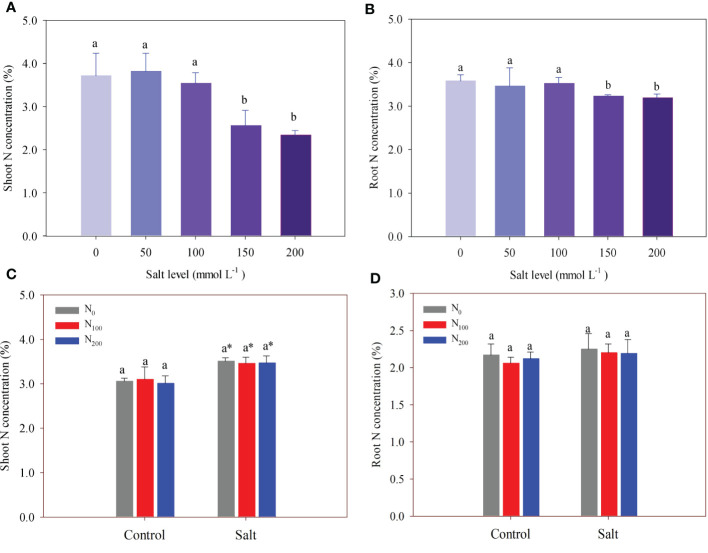
Alfalfa shoot **(A)** and root **(B)** nitrogen (N) concentration in different salt levels in experiment 1. Alfalfa shoot **(C)** and root **(D)** N concentration in different N supply and salt levels in experiment 2. Error bars represent ± SD of the mean. Different letters represent a significant difference among treatments (*p* ≤ 0.05). Asterisks refer to significant differences between salt levels at the same N fertilizer supply rates.

In experiment 1, salt addition did not change shoot and root N concentration, which was 3.98% on average until the salt level was above 100 mmol Na_2_SO_4_ L^–1^ (**Figures 3A, B**). Shoot N concentration decreased by 31.2% in alfalfa treated with 150 mmol Na_2_SO_4_ L^–1^ and by 37.2% in alfalfa treated with 200 mmol Na_2_SO_4_ L^–1^, compared with that in the control (**Figure 3A**). The difference observed in shoot N concentration between these two treatments was not statistically significant. Like shoot N concentration, root N concentration decreased by 9.7% in alfalfa treated with 150 mmol Na_2_SO_4_ L^–1^ and 10.8% in alfalfa treated with 200 mmol Na_2_SO_4_ L^–1^ compared with that in the control (**Figure 3B**).

In experiment 2, salt level significantly increased shoot N concentration by 11.7%–15.2% compared with treatments without salt but did not change root N concentration (**Figures 3C, D**). There was no difference in shoot N concentration among different N supply treatments, regardless of salt stress. The same result was found in root N concentration.

**Figure 4:**
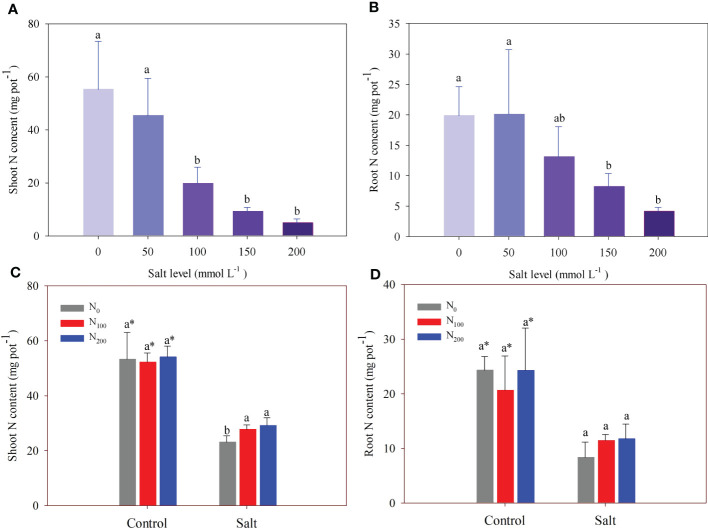
Alfalfa shoot **(A)** and root **(B)** nitrogen (N) content in different salt levels in experiment 1. Alfalfa shoot **(C)** and root **(D)** N content in different N supply and salt levels in experiment 2. Error bars represent ± SD of the mean. Different letters represent a significant difference among treatments (*p* ≤ 0.05). Asterisks refer to significant differences between salt levels at the same N fertilizer supply rates.

In experiment 1, there was no difference in shoot and root N content between the control and alfalfa treated with 50 mmol Na_2_SO_4_ L^–1^ (**Figures 4A, B**). However, salt addition significantly decreased shoot N content compared with that in the control by 64%, 83%, and 91% at salt levels of 100, 150, and 200 mmol Na_2_SO_4_ L^–1^, respectively (**Figure 4A**). In addition, root N contents of alfalfa treated with 100, 150 and 200 mmol L^–1^ Na_2_SO_4_ levels were 58%, 79%, and 66% significantly lower than that in the control, respectively (**Figure 4B**).

In experiment 2, N supply partially recovered the loss of shoot N content caused by salt addition (**Figures 1D–F**). Shoot N content was higher (10%–28%) in alfalfa treated with N supply than in alfalfa treated without N supply when they suffered the same salt stress (**Figure 4C**). In contrast, N supply did not change root N content (**Figure 4D**).

### 3.3 Root nodules and %Ndfa

**Figure 5:**
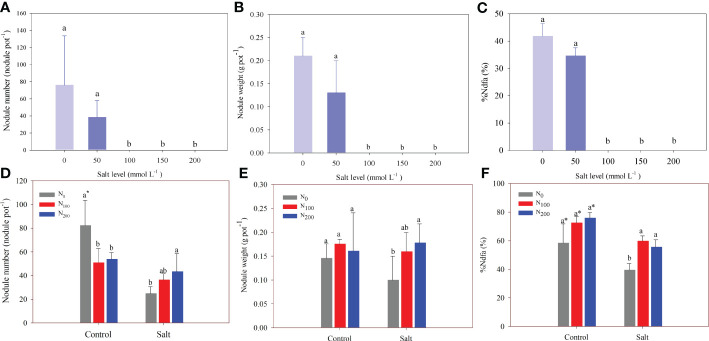
Alfalfa nodule number **(A)**, nodule weight **(B)**, and nitrogen derived from the atmosphere (%Ndfa) **(C)** in different salt levels in experiment 1. Alfalfa nodule number **(D)**, nodule weight **(E)**, and %Ndfa **(F)** in different nitrogen (N) supply and salt levels in experiment 2. Error bars represent ± SD of the mean. Different letters represent a significant difference among treatments (*p* ≤ 0.05). Asterisks refer to significant differences between salt levels at the same N fertilizer supply rates.

In experiment 1, nodule formation was inhibited when the salt level was above 100 mmol Na_2_SO_4_ L^–1^ (**Figures 5A, B**). There were 38 and 76 nodules per pot in the control and the alfalfa treated with 50 mmol Na_2_SO_4_ L^–1^, respectively, and nodule weight ranged from 0.13 to 0.21 g pot^–1^. In addition, the corresponding %Ndfa ranged from 35% to 42%, and amount of N fixed ranged from 18.08 to 23.72 mg pot^–1^ when the salt level was belove 50 mmol Na_2_SO_4_ L_–1_ (**Figure 5C**; **Table 1**). The N fixation efficiency showed the same result. There was no difference in number of root nodules, weight, %Ndfa, or amount of N fixed between the control and alfalfa treated with 50 mmol Na_2_SO_4_ L^–1^ (**Figure 5**; **Table 1**).

**Table 1:** Amount of nitrogen (N) fixed and N fixation efficiency of nodules in different salt levels in experiment 1.

Salt level (mmol Na_2_SO_4_ L^–1^)	Amount of N fixed (mg pot^–1^)	N fixation efficiency (mg g^–1^)
0	23.72 ± 10.66 a	107.44 ± 26.74 a
50	18.08 ± 0.71 a	119.62 ± 25.41 a
100	0 b	0 b
150	0 b	0 b
200	0 b	0 b

Values are means (n = 4) ± SD. Different letters represent a significant difference among different salt levels (p ≤ 0.05).

In experiment 2, the number of nodules decreased owing to salt stress only in the treatment with no N supply. Although salt stress did not change the nodule number, it did significantly decrease %Ndfa by 18%–33% compared with the alfalfa treated without salt stress. Salt addition significantly decreased the amount of N fixed by 41%–55%. A decrease of N fixation efficiency caused by salt stress was observed in alfalfa treated with a supply of 100 and 200 mg N kg^–1^ (**Table 2**). The number and weight of nodules significantly increased by 80% and 78%, respectively, in alfalfa treated with 200 mg N kg^–1^ compared with alfalfa treated without N supply and subjected to salt stress (**Figures 5D, E**). The %Ndfa was significantly higher by 53% and 42% in alfalfa treated with 100 and 200 mg N kg^–1^ than in alfalfa with no N supply (**Figure 5F**). The N supply improved the amount of N fixed by approximately 60% but had no effect on the N fixation efficiency of nodules (**Table 2**).

**Table 2:** Amount of nitrogen (N) fixed and N fixation efficiency of nodules in different N supply rates and salt levels in experiment 2.

Salt level	N supply rate (mg kg^–1^)	Amount of N fixed (mg pot^–1^)	N fixation efficiency (mg g^–1^)
Control	0	30.59 ± 6.21 a*	223.83 ± 97.47 a
	100	37.78 ± 2.36 a*	215.64 ± 11.15 a*
	200	41.13 ± 4.94 a*	288.54 ± 98.82 a*
Salt	0	13.72 ± 1.67 b	164.98 ± 72.75 a
	100	22.14 ± 1.98 a	145.41 ± 34.59 a
	200	22.06 ± 2.91 a	132.33 ± 51.83 a

Values are means (n = 4) ± SD. Different letters represent a significant difference at the same Na_2_SO_4_ level (p ≤ 0.05). Asterisks refer to significant differences between salt levels at the same N fertilizer supply rates.

### 3.4 Alfalfa quality

**Figure 6:**
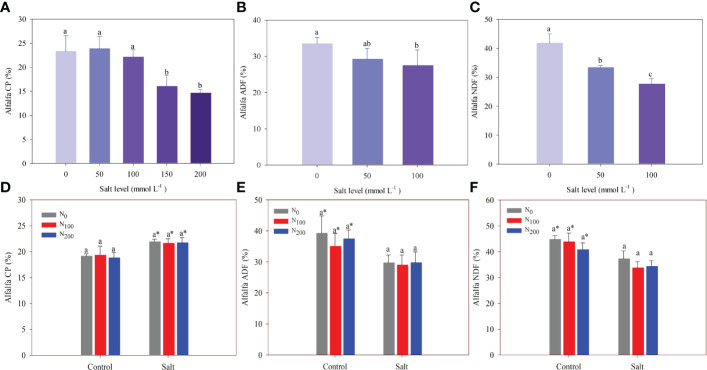
Alfalfa crude protein (CP) **(A)**, acid detergent fiber (ADF) **(B)**, and neutral detergent fiber (NDF) **(C)** in different salt levels in experiment 1. Incomplete ADF and NDF data owing to insufficient sample weight (shoot dry weight). Alfalfa CP **(D)**, ADF **(E)**, and NDF **(F)** in different nitrogen (N) supply and salt levels in experiment 2. Error bars represent ± SD of the mean. Different letters represent a significant difference among treatments (*p* ≤ 0.05). Asterisks refer to significant differences between salt levels at the same N fertilizer supply rates.

In experiment 1, salt addition did not change alfalfa CP, which was 23% on average until the salt level was above 100 mmol Na_2_SO_4_ L^–1^. Alfalfa CP significantly decreased by 31% and 37% in alfalfa treated with 150 and 200 mmol Na_2_SO_4_ L^–1^, respectively, compared with the control (**Figure 6A**). Alfalfa ADF and NDF decreased significantly with increasing salt levels. Compared with the control, ADF did not change in alfalfa treated with 50 mmol Na_2_SO_4_ L^–1^ but showed a significant decrease of 18% in alfalfa treated with 100 mmol Na_2_SO_4_ L^–1^ (**Figure 6B**). Salt addition significantly decreased the NDF of alfalfa by 20% and 34% in treatments of 50 and 100 mmol Na_2_SO_4_ L^–1^, respectively, compared with the control (**Figure 6C**).

In experiment 2, salt stress significantly increased CP by 11%–15% and decreased ADF and NDF by 15%–24% compared with alfalfa treated with the same N levels but without salt stress. The N supply did not change alfalfa CP content, which was 19.1% and 21.7% on average in the alfalfa treated without and with salt stress, respectively (**Figure 6D**). The same results were found for alfalfa ADF and NDF. The ADF was 37.2% and 29.5%, and NDF was 43.1% and 35.1% on average in the alfalfa treated without and with salt stress, respectively (**Figures 6E, F**).

## 4 Discussion

### 4.1 Response of alfalfa growth to salt stress and N supply

Salt stress had a negative effect on alfalfa growth and biomass in experiment 1. Additionally, experiment 2 also confirmed the results and showed N supply alleviated the negative effects of salt stress. Like chickpeas, alfalfa can bear a light salt stress that was less than 100 mmol Na_2_SO_4_ L^–1^ (Qurashi and Sabri, 2013). One reason for this could be the accumulation of soluble sugars and proline in plants experiencing light salt stress, which facilitates the maintenance of the cytoplasmic osmotic pool for growth stabilization and cellular metabolism in plants (Farooq et al., 2017). However, once salt concentration exceeds the critical level, plant growth is inhibited by salt toxicity (Samineni et al., 2011). The results show that the height, stem diameter, and branching number of alfalfa significantly decreased when the salt level was more than 100 mmol Na_2_SO_4_ L^–1^ (**Figures 1A–C**). These responses were attributed to the decline in the water potential of tissue, which results in the closure of stomata and sequentially decreases photosynthesis, resulting in reduced growth (Garg and Manchanda, 2009; Garg and Bhandari, 2016). Root growth is more vulnerable to salt stress than shoot growth for safflower (Kaya, 2003). Our results did not support this difference between root and shoots for alfalfa, as they showed a similar response to salt stress (**Figures 2C, D**).

Alfalfa growth inhibited by salt stress was partially overcome by treatments with N supply (**Figures 1D–F**). This finding was consistent with previous studies in which soybeans suffering salt stress could still achieve high plant biomass when supplied with 120 mg N kg^–1^ soil in a pot experiment (Abdel Wahab and Abd Alla, 1995). Sikder et al. (2020) found that N supply increases plant water status, photosynthetic pigment synthesis, and gas exchange attributes, and further improves plant growth in salt-stressed conditions. In addition, N supply also increases the activity of defense enzymes and decreases salt ion concentrations to alleviate the negative effect on plant growth of legumes suffering salt stress (Kirova, 2020; Hashemi et al., 2022). In this study, however, N supply did not have any stimulation for alfalfa growth except where salt stress was induced (**Figures 2C, D**). This is consistent with the results of He et al. (2018), who found no significant difference in the shoot growth of alfalfa among treatments comprising different N application rates when plants were not suffering salt stress in the field.

### 4.2 Response of alfalfa N uptake to salt stress and N supply

Salt accumulation in the rhizosphere causes a nutritional imbalance in plants (Miransari and Smith, 2007), including the inhibition of N adsorption (Rabie and Almadini, 2005). Our results showed that alfalfa shoot and root N concentrations significantly decreased when the salt level was above 150 mmol Na_2_SO_4_ L^–1^ (**Figures 3A, B**), but the shoot and root N contents were more sensitive to salt addition than concentrations (**Figures 4A, B**). Previous studies show decreased biomass accumulation leads to a higher nutrient concentration in plants because of the concentration effect (Jarrell and Beverly, 1981; Li et al., 2010). We suspect that this is why shoot N concentrations became higher when the alfalfa suffered salt stress in experiment 2 (**Figures 3C, D**; **Figures 4C, D**). The N supply significantly increased shoot and root N contents through accelerating alfalfa growth in salt-affected soil (**Figures 4C, D**). This indicates that salt stress induced N deficiency in the alfalfa in this study. The N supply improved plant N nutrition when alfalfa suffered salt stress, in line with previous studies (Wortmann et al., 2000; Abbasi et al., 2011).

### 4.3 Response of alfalfa N fixation to salt stress and N supply

Salt stress significantly suppresses nodulation formation in legumes (Li et al., 2021). For instance, salt stress has been found to substantially decrease the activity and density of nodules in the pigeon pea by two to three times (Garg and Manchanda, 2008). The nodule number and weight in alfalfa were also significantly reduced by increasing salt levels in this study (**Figures 5A–C**). The reasons for this due to salt stress could include poor root growth, fewer available photosynthates, fewer hairs, and lower respiration rate (Manchanda and Garg, 2008; Cornacchione and Suarez, 2015; Díaz et al., 2018). These factors are necessary for nodule formation, and the effects of salt stress on them further lead to the negative impacts on the colonization of rhizobia in the root (Del Pilar Cordovilla et al., 1999; Arora, 2015). Furthermore, salt stress decreased nodule number but not nodule weight in alfalfa treated without N supply (**Figures 5D, E**). This indicates that salt stress inhibited nodule emergence rather than nodule growth. In addition, N fixation still requires N from the soil for the early stage of nodule formation (Bordeleau and Prevost, 1994; Lindström and Mousavi, 2020). Thus, N supply did not increase alfalfa nodule formation and the amount of fixed N owing to there being sufficient soil N in the no-salt-stress condition (**Figures 5D–F**; **Table 2**). However, as discussed above, the N fixation processes of alfalfa were disturbed by salt stress. Thus, it was not unexpected that N supply increased the nodule number and weight of alfalfa in salt-affected soil in this study because N supply provides the starting N for nodules and enhances the N fixation efficiency of rhizobia through increasing the activity of nitrogenase and nitrate reductase when legumes suffer salt stress (Abdel Wahab and Abd Alla, 1995; Liu et al., 2022). This result aligns with previous studies (Undersander, 2011; Elgharably and Benes, 2021). Salt stress also reduced the N fixation efficiency of nodules, indicating that N fixation processes were disturbed by the salt.

### 4.4 Response of alfalfa quality to salt stress and N supply

Salt stress decreased the CP, ADF, and NDF of alfalfa in this study (**Figures 6A–C**), which is consistent with the results of Yan et al. (2005). Similar results have also been found in marvel grass (Kumar et al., 2018). As per the discussion above, salt addition decreases CP concentration in alfalfa because of the inhibition of N uptake and N fixation (El-Sharkawy et al., 2017). The low cell wall and lignin concentrations caused by salt addition are responsible for the decreased ADF and NDF (Oliveira et al., 2020). A meta-analysis showed that the CP concentration of alfalfa increases with N supply, which is due to the higher activity of key enzymes for N metabolism (Geisseler et al., 2010; Wan et al., 2022). However, N supply did not change the CP concentration of alfalfa in this study (**Figure 6D**). This may be explained by the alfalfa growing so fast that it caused the dilution effect (Jarrell and Beverly, 1981). Our results showing that alfalfa ADF and NDF were not responsive to N supply (**Figures 6E, F**), are also consistent with the results of Cherney et al. (1994).

## 5 Conclusion

Salt stress significantly decreased not only alfalfa biomass and quality but also N fixation through inhibiting nodule formation and reducing N fixation efficiency. This may be due to poorer plant N nutrition, as our results confirmed that N supply can partially offset the inhibition of growth and N fixation caused by salt stress. Thus, optimal N fertilizer application is essential to alleviate loss of growth and N fixation in salt-affected soils.

## Data availability statement

The original contributions presented in the study are included in the article/supplementary material. Further inquiries can be directed to the corresponding author.

## Author contributions

WW carried out the experiment, analyses data and wrote the manuscript; QL, CZ, and KL helped carry out the experiment. ZS and YL supervised the research; HL helped perform the analysis with constructive discussions and revised manuscript. All authors contributed to the article and approved the submitted version.
